# Computed Tomography Findings in Non-Obstetric Vulvar Hematoma: A Case Report

**DOI:** 10.21980/J8194H

**Published:** 2024-10-31

**Authors:** Eleanor M Birch, Theodore McClean, Scott Szymanski

**Affiliations:** * Madigan Army Medical Center, Department of Emergency Medicine, Joint Base Lewis-McChord, WA

## Abstract

Non-obstetric vulvar hematoma is a rare but clinically important diagnosis in the emergency department for which there is no consensus on optimal diagnosis or management. We present a case of non-obstetric vulvar hematoma that occurred after minimal trauma in a young, otherwise healthy woman who presented with labial swelling after consensual digital penetration, initially managed conservatively but ultimately requiring surgical drainage. Although a rare presentation in the emergency department, prompt identification, diagnosis, and management of vulvar hematoma is crucial to appropriately treat complications including pain, hemodynamically significant hemorrhage, urinary obstruction, and soft tissue necrosis.

**Topics:**

Vulvar hematoma, pelvic trauma, women’s health, CT (computed tomography) angiography.

[Fig f1-jetem-9-4-v6] [Fig f2-jetem-9-4-v6]

**Figure f1-jetem-9-4-v6:**
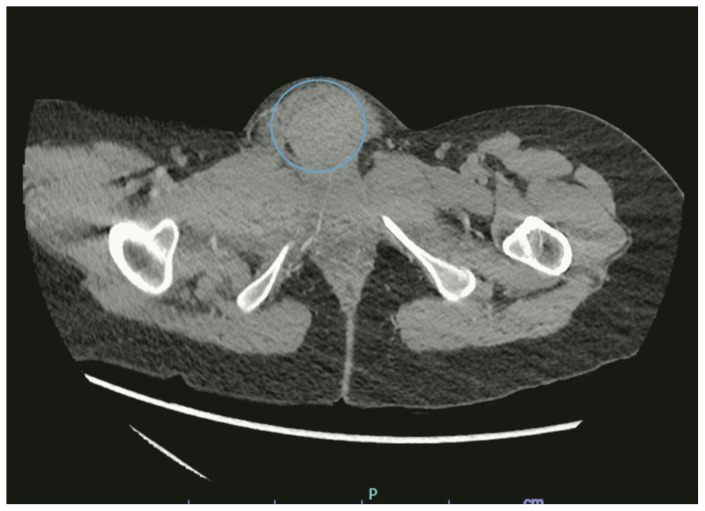


**Figure f2-jetem-9-4-v6:**
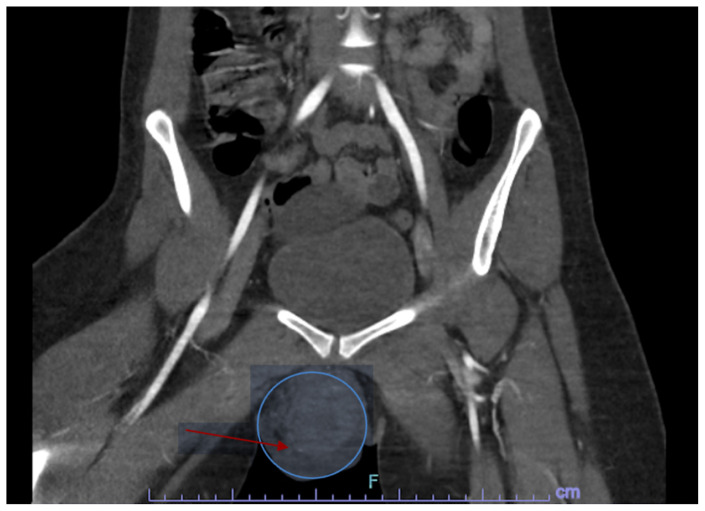


## Brief introduction

While common in the obstetric population, non-obstetric vulvar hematomas are rarely reported in the literature. When seen, non-obstetric vulvar hematomas are most often related to significant vulvar trauma, such as saddle injury or sexual assault.^1^,^2^ Spontaneous hematomas may also occur as the result of spontaneous rupture of the iliac or pudendal artery.^3^,^4^ Prompt recognition and management of vulvar hematomas is critical in the emergency department because they can result in hemodynamically significant hemorrhage, urinary obstruction, and soft tissue necrosis. We present a case of non-obstetric vulvar hematoma in a patient without significant trauma history to highlight the imaging findings and management of this condition in the Emergency Department setting.

## Presenting concerns and clinical findings

A 25-year-old woman, gravida two para two, presented to the emergency department with acute onset vulvar swelling. Just prior to onset she had experienced consensual digital vaginal penetration with her partner, but she denied other penetrative intercourse or vulvovaginal trauma. She had no preceding pain, redness, or swelling, and no history of similar symptoms in the past. She denied any possible allergen exposure. She had no vaginal discharge, bleeding, dysuria, or other urinary symptoms. She was monogamous with a single male partner and had no history of sexually transmitted infection, labial cyst, or abscess. Initial vital signs revealed a heart rate of 82, blood pressure of 114/71, and an oxygen saturation of 99%. On physical exam, the patient was noted to have a 15 cm by 10 cm firm mass of her right labia with severe tenderness to palpation. The physical exam was otherwise unremarkable, without evidence of contusion, laceration, ecchymosis, or other signs of trauma to the head or neck, extremities, trunk, or on external exam of the vulva or rectum.

## Significant findings

Bedside ultrasound was first used to evaluate for evidence of abscess or cyst formation. Ultrasound demonstrated a hypoechoic area within the right labia without evidence of a cyst or abscess wall. Based on these findings, an angiogram CT of the pelvis was obtained which revealed a vulvar hematoma with evidence of active arterial extravasation. In both the coronal and axial view, there is an asymmetric area of isodensity in the right labia representing a hematoma (blue circled area). Angiography may show areas of active extravasation, which appears as hyperdensity within the area of hematoma (see red arrow in coronal plane).

## Patient course

The patient’s pain was treated with hydromorphone followed by pain-dosed ketamine. A pediatric-sized foley catheter was placed due to concern for urinary obstruction. Obstetrics and gynecology were consulted, and the patient was admitted to the gynecology service for observation and pain control. Her initial hemoglobin was 13.3 g/dL. Her hemoglobin dropped to 11.3 g/dL on hospital day one and stabilized by hospital day two. Her hematoma did not continue to expand, and her condition was managed conservatively, without surgical intervention or embolization, until discharge on hospital day four. Due to continued swelling and reported pain, as well as concern for soft tissue necrosis, the patient underwent hematoma drainage with removal of 200 cc of blood clot three weeks after her initial presentation. She recovered well and began pelvic floor physical therapy and treatment for symptoms of vaginismus.

## Discussion

This case illustrates a rare presentation of a non-obstetric vulvar hematoma without significant trauma history. In the obstetric setting, vulvar hematomas are estimated to occur in 1:500 to 1: 12500 deliveries. Most small hematomas can be managed conservatively with compression and analgesia. Infrequently, in an estimated 1:1000 vaginal deliveries, they require surgical intervention,^5^,^6^,^7^ Most reported cases of nonobstetric hematomas have occurred in the setting of vulvar trauma, including saddle injuries, assault, or other blunt trauma. These cases are also most often managed conservatively, though rarely they require resuscitation with blood transfusion, arterial embolization, or surgical ligation and evacuation of clot. 8, 9, 10, 11 This case represents an atypical presentation of an already rare condition for which the diagnosis was not initially clear on presentation. It also demonstrates the fundamentals of management, which include stabilization and monitoring, pain control, treatment of urinary retention, appropriate imaging, and early consultation with surgical specialists.

Several other etiologies for vulvar and labial swelling were also considered during the patient’s evaluation. Bartholin gland abscesses and cysts, folliculitis, and vulvar varicosities may all present with similar vulvar pain and edema. The history and progression of swelling can aid with differentiating these etiologies; however, imaging including ultrasound and angiography CT can also provide important differentiating information. Ultrasound as an initial tool may help identify a thick cyst wall or heterogenous hypoechoic material consistent with a loculated abscess. Angiography CT provides the ability to identify culprit vessels when there is concern for active extravasation, as with this patient’s rapidly expanding edema. While there is no consensus on a gold standard for imaging vulvar hematoma, it is notable that arterial embolization is an increasingly utilized tool for the management of pelvic trauma in general, including vulvar hematoma. 3, 4, 12,13 Angiography CT can provide valuable information regarding the source of bleeding in vulvar hematoma, which often stems from a branch of the pudendal artery. Early inclusion of vulvar hematoma on the differential of a patient presenting with vulvar edema and pain may help guide appropriate imaging because angiography CT can better identify a source of arterial bleeding in hematomas requiring intervention.^14^, ^15^ This is particularly relevant for patients with evidence of hemodynamic instability or those requiring blood transfusion, or in patients with spontaneous, atraumatic vulvar hematomas, for whom spontaneous vessel or aneurysm rupture may be a more likely etiology for their bleeding. 3,4

Ultimately, prompt recognition of vulvar hematoma as a source of labial swelling, even without a significant trauma history, is vital for appropriate imaging, management, and disposition of patients presenting with this condition.

## Supplementary Information








