# Supplementary figures and images for: Computed Tomography Findings in Non-Obstetric Vulvar Hematoma: A Case Report
Source: J Educ Teach Emerg Med. 2024 Oct 31;9(4):V6–9. doi: 10.21980/J8194H (PMC11537726; doi:10.21980/J8194H)

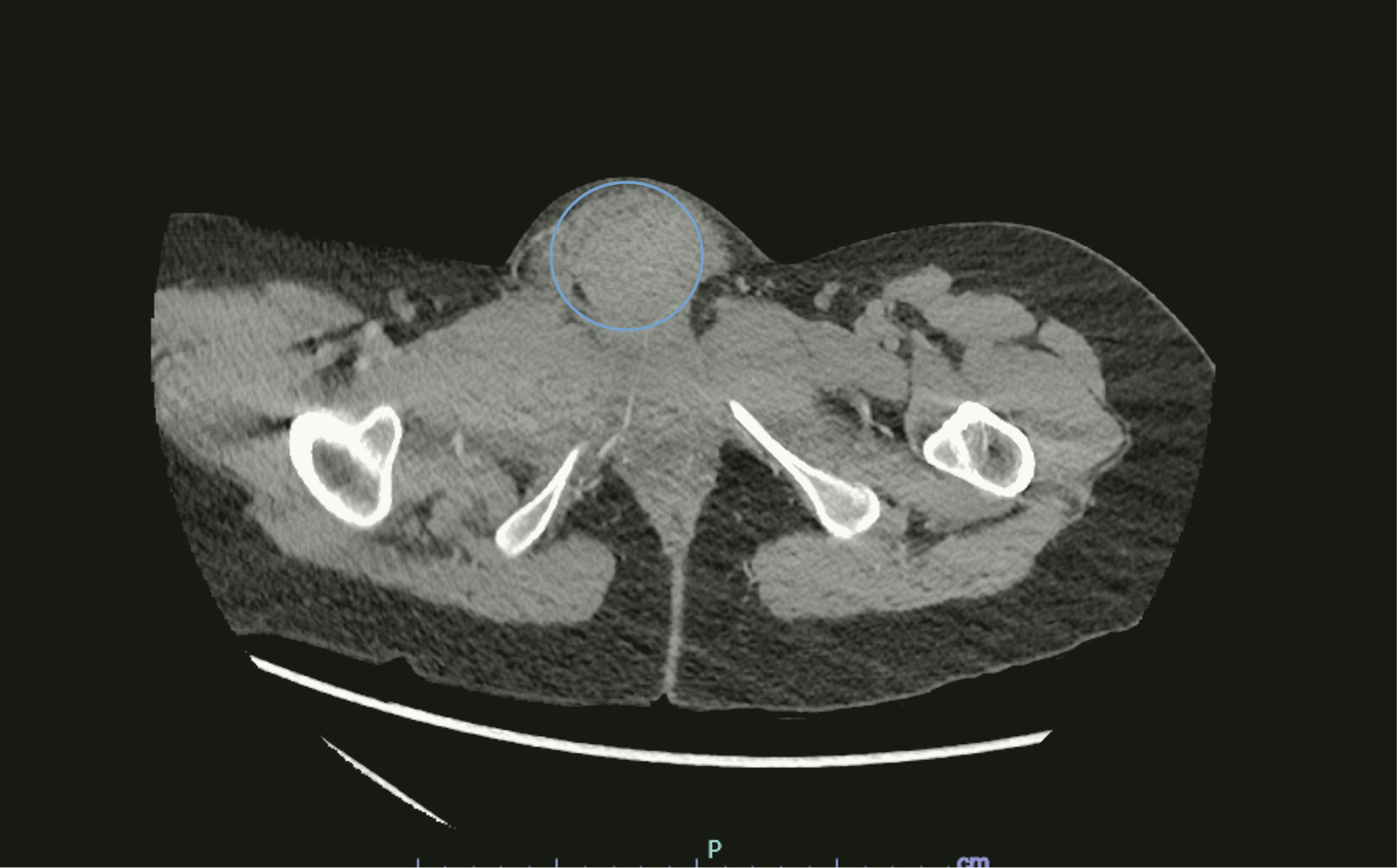

Supplement: Supplementary file 1 [file 9-4-V6-supp1.png]

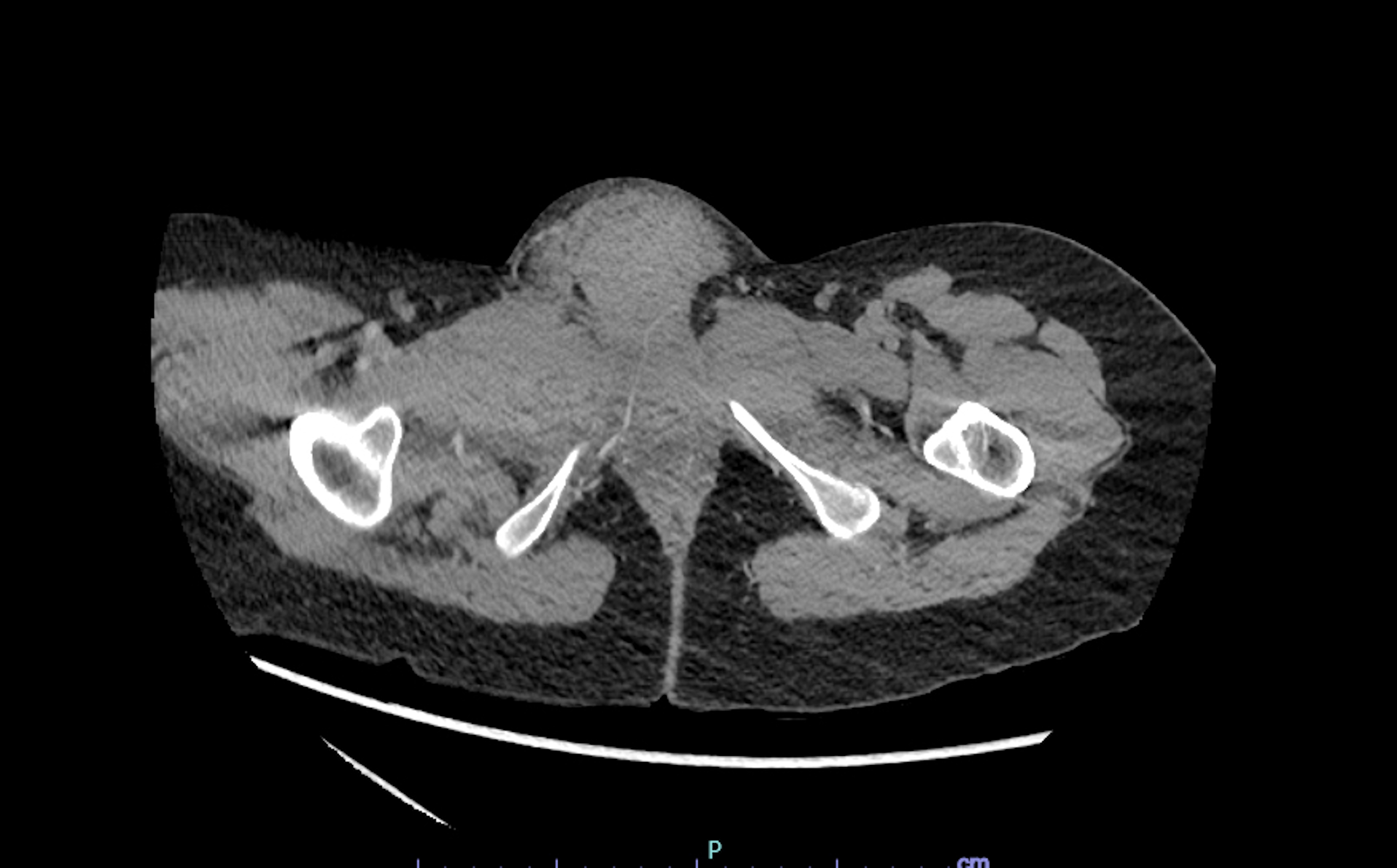

Supplement: Supplementary file 2 [file 9-4-V6-supp2.jpg]

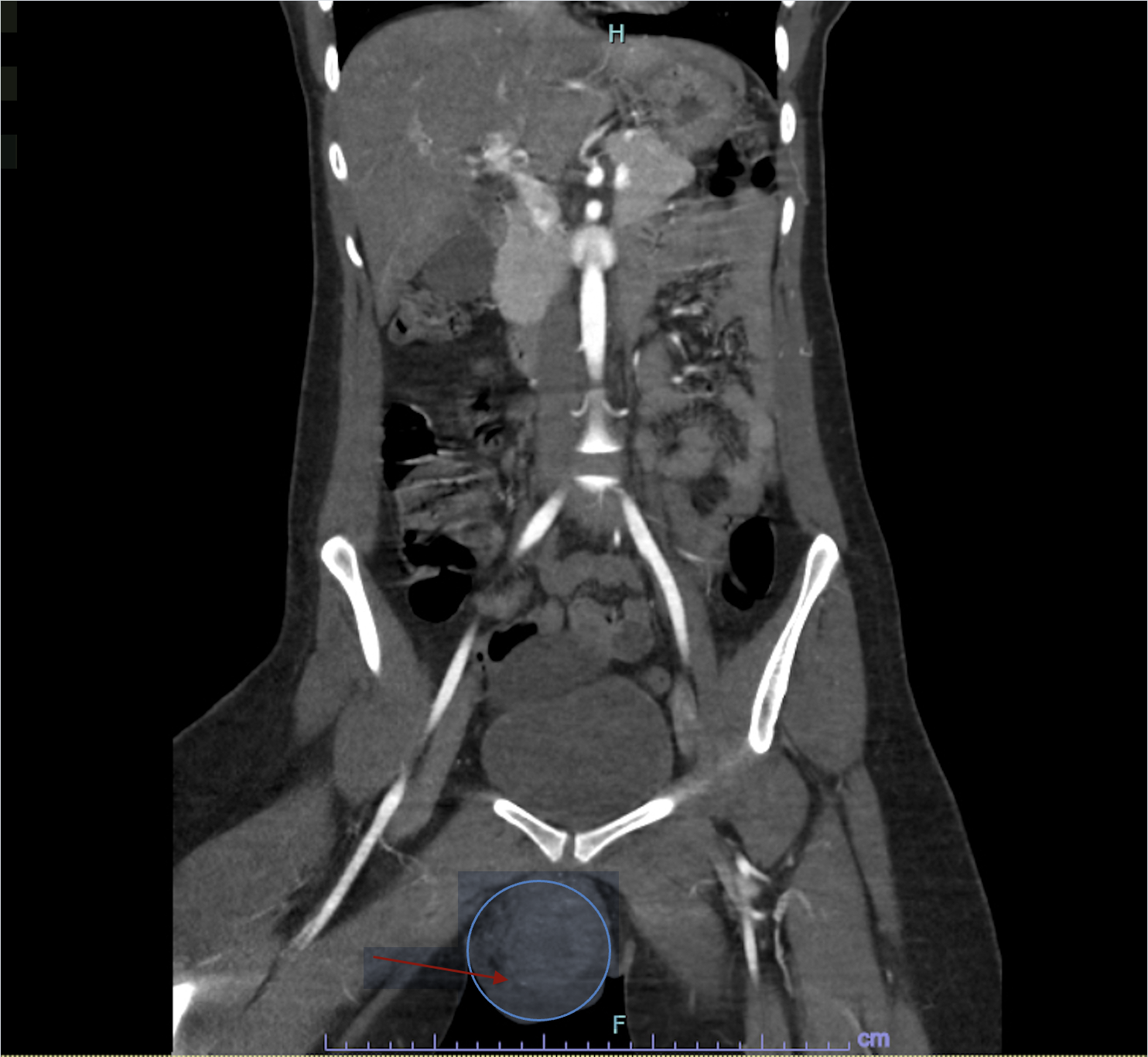

Supplement: Supplementary file 3 [file 9-4-V6-supp3.png]

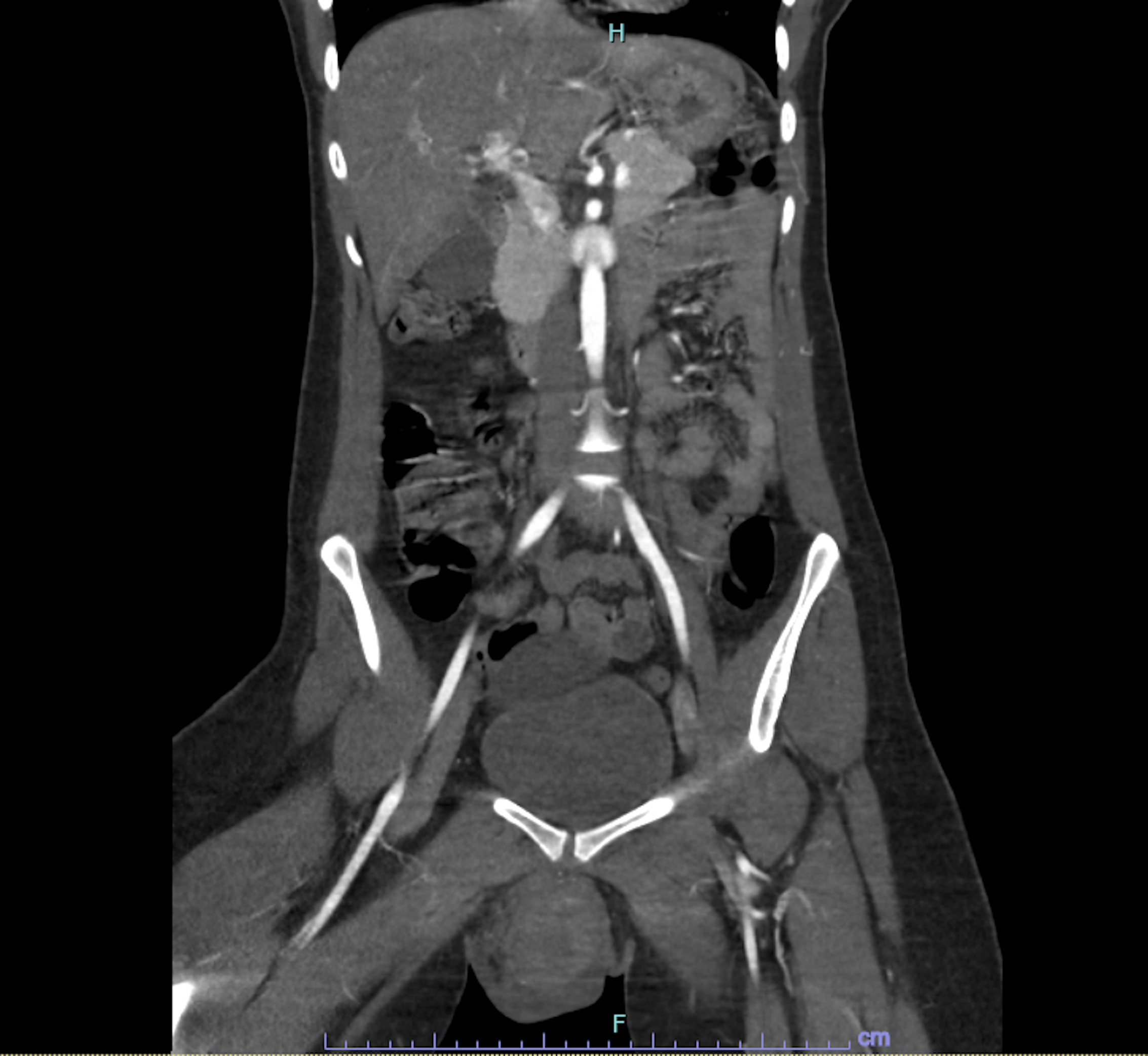

Supplement: Supplementary file 4 [file 9-4-V6-supp4.jpg]
